# Myticalins: A Novel Multigenic Family of Linear, Cationic Antimicrobial Peptides from Marine Mussels (*Mytilus* spp.)

**DOI:** 10.3390/md15080261

**Published:** 2017-08-22

**Authors:** Gabriele Leoni, Andrea De Poli, Mario Mardirossian, Stefano Gambato, Fiorella Florian, Paola Venier, Daniel N Wilson, Alessandro Tossi, Alberto Pallavicini, Marco Gerdol

**Affiliations:** 1 Scuola Internazionale Superiore di Studi Avanzati, via Bonomea 265, Trieste 34136, Italy; galeoni@sissa.it; 2 Department of Life Sciences, University of Trieste, Via Giorgieri 5, Trieste 34127, Italy; ANDREA.DEPOLI@studenti.units.it (A.D.P.); mmardirossian@units.it (M.M); STEFANO.GAMBATO@phd.units.it (S.G.); florian@units.it (F.F); atossi@units.it (A.T.); 3 Genzentrum, Ludwig Maximilian Universität, Feodor Lynen Straβe 25, München 81377, Germany; wilson@genzentrum.lmu.de; 4 Department of Biology, University of Padova, Via Bassi 58/B, Padova 35131, Italy; paola.venier@unipd.it

**Keywords:** antimicrobial peptide, innate immunity, *Mytilus galloprovincialis*, proline-rich, hypervariability

## Abstract

The application of high-throughput sequencing technologies to non-model organisms has brought new opportunities for the identification of bioactive peptides from genomes and transcriptomes. From this point of view, marine invertebrates represent a potentially rich, yet largely unexplored resource for *de novo* discovery due to their adaptation to diverse challenging habitats. Bioinformatics analyses of available genomic and transcriptomic data allowed us to identify myticalins, a novel family of antimicrobial peptides (AMPs) from the mussel *Mytilus galloprovincialis*, and a similar family of AMPs from *Modiolus* spp., named modiocalins. Their coding sequence encompasses two conserved N-terminal (signal peptide) and C-terminal (propeptide) regions and a hypervariable central cationic region corresponding to the mature peptide. Myticalins are taxonomically restricted to Mytiloida and they can be classified into four subfamilies. These AMPs are subject to considerable interindividual sequence variability and possibly to presence/absence variation. Functional assays performed on selected members of this family indicate a remarkable tissue-specific expression (in gills) and broad spectrum of activity against both Gram-positive and Gram-negative bacteria. Overall, we present the first linear AMPs ever described in marine mussels and confirm the great potential of bioinformatics tools for the *de novo* discovery of bioactive peptides in non-model organisms.

## 1. Introduction

Antimicrobial peptides (AMPs) are fundamental effector molecules of the humoral innate immune system that are widespread in animals and contribute to the first line of defense against pathogens. Expansion and diversification of both innate immune receptors and effectors is a common finding in animals, especially in invertebrate organisms that lack an adaptive immune system [1,2]. AMP research in mollusks is indissolubly linked to marine mussels (*Mytilus* spp.) since the mid-1990s, when active hemolymph fractions were used to purify several short cysteine-rich peptides, belonging to the defensin, mytilin, myticin and mytimycin families [3,4]. These AMPs are all expressed at high levels in hemocytes, circulating cells that are considered to be major players in molluscan immunity, and are all characterized by multiple disulfide bridges which ensure a precisely folded, stable structure for the mature peptides that are compact, cationic and amphipathic in nature. Although more recent reports have provided evidence for the presence of AMPs in other mussel tissues [5,6,7], a finding in line with the emerging role of mucosal immunity in bivalves [8,9], all currently known mussel AMPs can be placed within the cysteine-rich category based on the conventional structural classification of AMPs [10].

It is, in fact, rather puzzling that no AMPs belonging to the two other major and widespread structural categories, namely linear/α-helical AMPs and AMPs enriched in specific amino acids has ever been reported in *Mytilus* spp. and other mytiloids. Reports from other molluscan species are however still quite scant, possibly due to poor sequence homology with known AMPs of other phyla and the consequently difficult identification by sequence similarity-based methods [11].

As far as bivalve mollusks are concerned, the most remarkable case is certainly that of molluscidins, short and likely unstructured AMPs characterized by multiple dibasic residue repeats, identified in the gills of oysters (Bivalvia) and abalones (Gastropoda) [12,13]. Although the two reported molluscidins display similar length and amino acid composition, the lack of structural data for the abalone gene and the apparent absence of similar sequences in the available genomes of other mollusks [14,15] do not permit the assessment with any certainty as to whether these two gene products are orthologous or the product of convergent evolution. Moreover the organization of their protein precursors is quite unusual since it is devoid of signal peptide and an anionic pro-region. In any case, both molluscidins displayed a similarly broad spectrum of activity against bacteria, but were inactive against *Candida* spp. Furthermore, the inducibility of the oyster peptide upon injection of *Vibrio* sp., supports the involvement of linear cationic peptides in the molluscan immune defense.

Another example is provided by CgPrps (proline- and arginine-rich peptides) identified in the hemocytes of the Pacific oyster *Crassostrea gigas*. Although these peptides did not display any significant antimicrobial activity by themselves, they were able to synergistically enhance the activity of defensins co-occurring in stimulated hemocytes [16]. The only other account of AMPs devoid of cysteine residues in mollusks is related to *Rapana venosa*, a marine snail where multiple cationic Pro-rich AMPs with no similarity with any publicly available sequence have been isolated from the hemolymph [17].

However, linear cationic AMPs are not rare in other invertebrate taxa, arthropods in particular. Crab arasins, [18] hyastatins [19] and shrimp penaeidins [20,21] are highly diverse AMPs, all rich in Pro and Arg residues and only seldom associated with an additional domain bearing disulfide bridges. Metalnikowins from the hemipteran *Palomena frasina* [22], drosocins and metchnikowins from *Drosophila* [23,24], formaecins from the ant *Myrmecia gulosa* [25], oncocins and pyrrhocoricins from the sap-sucking-bug *Pyrrhocoris apterus* [26,27], lebocins from the silkworm *Bombyx mori* [28], astacidins from the freshwater crayfish *Procambarus clarkii* [29] and the large family of Pro-rich apidaecins from the honeybee *Apis mellifera* [30], are just some other examples of linear AMPs from nonmarine invertebrate organisms.

Taking into account the large disparity in the available literature related to linear AMPs in ecdysozoan and non-ecdysozoan protostomes, we developed an analytical pipeline to efficiently identify AMP-encoding transcripts in *M. galloprovincialis,* an organism with an abundant repertoire of defense peptides. This transcriptome-based approach relied on the detection of sequences encoding short secreted peptides with chemical-physical properties similar to other known cationic AMPs. The applied analysis did not rely on significant sequence similarity with other AMPs or the presence of known protein domains, thus overcoming inherent difficulties of routine bioinformatics methods in the detection of novel AMPs.

Overall, we could identify and characterize a large new family of potential AMPs, proposed here as myticalins, mainly expressed in mussel gills as prepropeptides. Their considerable diversity, both at the genomic DNA and mRNA level, suggests that these sequences are subject to diversifying evolution. The antimicrobial properties of seven chemically synthesized myticalins, were confirmed in vitro against a broad range of Gram-positive and Gram-negative bacteria.

## 2. Results and Discussion

### 2.1. Myticalins Pertain to a Multigenic Family

Following an in silico AMP discovery approach, we identified a transcript encoding a precursor protein targeted to the canonical secretory pathway. The signal peptide for secretion was followed by a highly positively charged region rich in proline, arginine and tyrosine residues arranged as PRY repeats, and by a negatively charged C-terminal region rich in aspartic and glutamic acid residues. These two regions with opposite charge were separated by a dibasic lysine/arginine proprotein convertase cleavage site. The chemical-physical properties and amino acid composition of the central region denoted a high antimicrobial potential, suggesting that it might correspond to a mature peptide released upon secretion of its precursor polypeptide into the extracellular space and further propeptide removal. Due to the absence of predictable secondary structure and its highly cationic character, the putative mature peptide was provisionally named myticalin, acronym for *Mytilus* cationic linear AMP.

**Figure 1:**
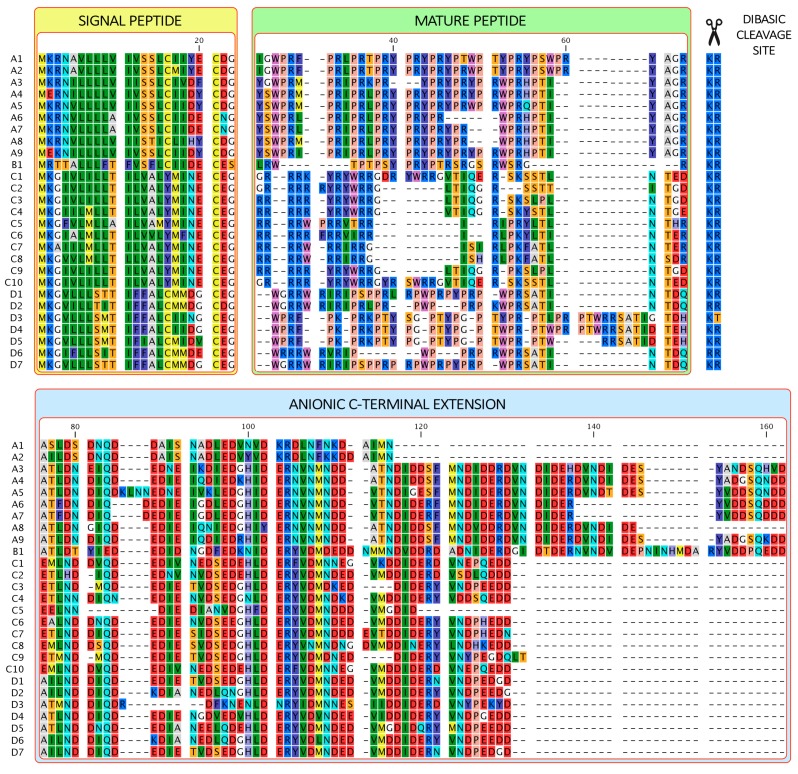
Multiple sequence alignment of all myticalin precursor sequences identified in *Mytilus* spp. Signal peptide, mature peptide region, dibasic propeptide cleavage site and anionic C-terminal extension regions are highlighted. Amino acids are colored according to their chemical-physical properties. All the reported sequences pertain to the *Mytilus edulis* species complex (*M. galloprovincialis*, *M. edulis*, *M. trossolus* and *M. chilensis*), with the exception of myticalin A1, A2, A6, A7, C4 and C7, which were identified in *M. californianus*.

Subsequent BLAST searches in the available mussel transcriptomes revealed many additional myticalin-like sequences, sharing high homology with each other mostly in the N-terminal signal peptide or in the C-terminal anionic extension, but only seldom in the central mature peptide region. The multiple alignment of the 28 full-length sequences identified in *Mytilus* spp. revealed some peculiar features shared by all myticalins (Figure 1). In the first place, the entire signal peptide region was well conserved in all sequences (50% majority-rule consensus sequence = MKGXXLLLLTIXXALCMIXECEG), as often reported for other AMPs [31,32,33] and toxins [34,35]. The Lys-Arg dibasic site was conserved in nearly all sequences, whereas the anionic C-terminal extension of variable length (35–75 amino acids) displayed moderate conservation.

Notably, the central, mature peptide region was found to be extremely variable, both in terms of length (ranging from 23 to 42 residues) and amino acid composition. Notwithstanding their limited sequence homology, all the putative mature peptide regions shared a high isoelectric point (>11) and >10% content of arginine residues. Frequently, the cleavage of the anionic extension was predicted to result in a Gly/Arg dipeptide at the C-terminus of the central region, which is expected to lead to the C-terminal amidation of the amino acid residue N-terminal to Gly, by the combined action of carboxypeptidase E (CPE) and peptidylglycine α-amidating monooxygenase (PAM). This is a common feature of AMPs that serves to increase cationicity and reduce degradation, and may have functional consequences [36].

Further analysis of the amino acid content of myticalins and reciprocal similarity observed in the multiple sequence alignment reported in Figure 1 allowed the classification of these AMPs into four subfamilies: (i) A, rich in Arg, Pro and Tyr residues (>10% each); (ii) B, containing a single member, rich in Pro, Arg and Thr residues; (iii) C, rich in Arg, but nearly devoid of Pro residues; (iv) D, rich in Pro Arg and Trp (or Thr) residues. Myticalins were named using a two-symbol code, with a letter indicating the group, and a number distinguishing the group member. Focusing on the physicochemical properties of the inferred mature peptide, the proposed classification does not consider the species of origin (owing to genetic introgression a specific myticalin may occur in more than one species of *M. edulis* sp. complex), minor polymorphisms occurring in the signal peptide/propeptide regions or synonymous nucleotide substitutions. The full length myticalin sequences identified in this study have been deposited in GenBank with the accession IDs MF432160–MF432186.

**Figure 2:**
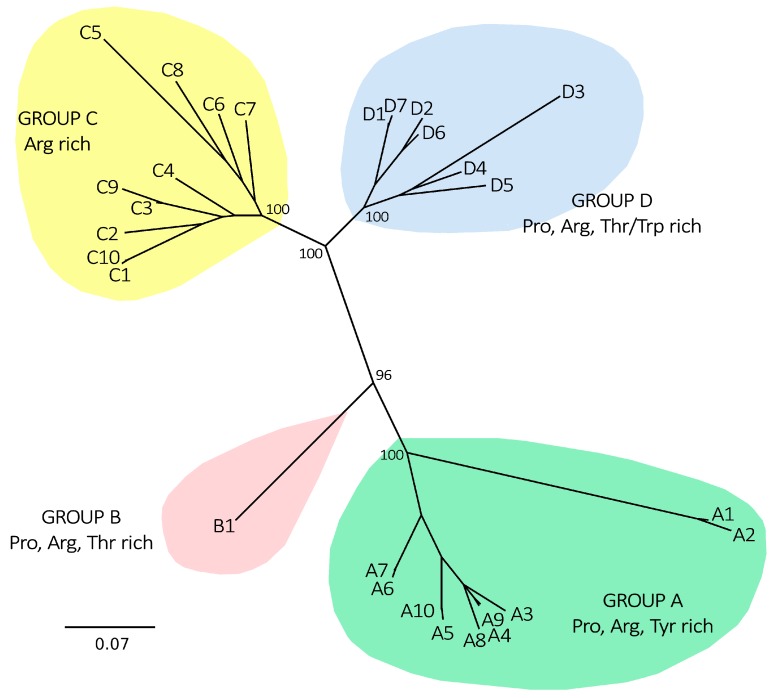
Unrooted Bayesian phylogenetic tree summarizing the relationships among myticalins. The tree topology is based on the multiple sequence alignment of the nucleotide sequences encoding the precursor proteins of myticalins, with the hypervariable mature peptide region removed. Codon triplets were preserved and the tree topology was calculated based on a codon w variation model of molecular evolution. Only posterior probability support values for the major nodes are displayed for clarity’s sake. All the sequences displayed in the tree pertain to the *Mytilus edulis* species complex (*M. galloprovincialis*, *M. edulis*, *M. trossolus* and *M. chilensis*), with the exception of myticalin A1, A2, A6, A7, C4 and C7, which were detected in *M. californianus*.

Such categorization nearly perfectly mirrored the phylogenetic relationship of sequences, based on their alignable portions (i.e., not taking into account mature peptide divergent regions), and the three major groups (A, C and D) were supported by extremely significant posterior probability values (Figure 2). Myticalin B1, clustered apart from the three aforementioned myticalin subfamilies, in agreement with its peculiar amino acid composition. Despite their significant divergence, myticalins C and D were more closely related to each other than with myticalins A or B. The divergence between the *M. californianus* myticalin A1/A2 and the other group A myticalins can be linked to the unusually short C-terminal extension of the former (Figure 1).

### 2.2. Gene Structure and Interindividual Variability

**Figure 3:**
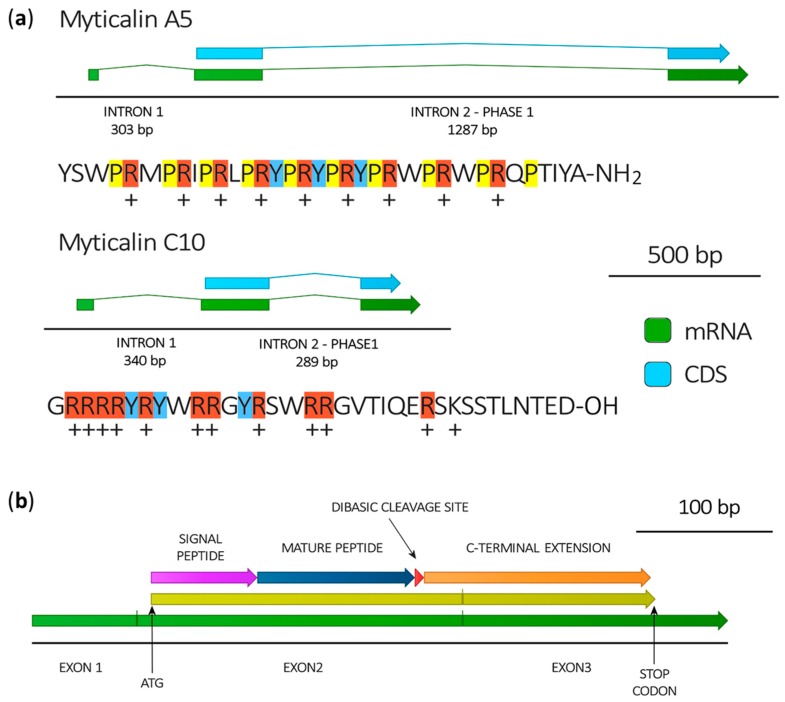
(**a**) Gene structure of myticalin A5 and myticalin C10, deduced by the alignment of *de novo* assembled transcripts with the draft reference genome of *Mytilus galloprovincialis* [37]. The mature peptides generated by the post-translational processing of the prepropeptides encoded by the two genes are also indicated, highlighting proline (yellow), arginine (red) and tyrosine (blue) residues and indicating positive net charges at pH = 7; (**b**) Typical organization of myticalin mRNAs.

The exon/intron architecture of myticalin genes was investigated in the publicly available draft genome of *M. galloprovincialis* [37]. However, the high fragmentation of the genome impaired a detailed study of the genomic context, association with neighboring genes and myticalin promoter regions. Nevertheless, we could retrieve the full-length sequence of two genes, encoding myticalin A5 and C10, each one composed by three exons and two introns. Partial genomic data for other myticalins fully confirmed the consistency of exon/intron boundaries for all the members of this family. In detail, the first short exon exclusively contains the 5′UTR region (Figure 3). The first relatively short intron (303 bp or 340 bp) interrupts the 5′UTR region right before the ATG start codon, which is therefore located at the 5′ end of exon 2. Exon 2 encompasses the entire signal peptide, the complete mature peptide region, the dibasic propeptide cleavage site and the first few residues of the anionic extension. The second intron is in phase 1 and displays a marked length difference between the myticalin C10 and A5 genes (289 bp and 1287 bp, respectively). The third and final exon encodes the remaining part of the precursor C-terminal extension, and includes the 3′UTR region up to the polyadenylation signal (Figure 3).

The simultaneous availability of a completely assembled genome and of the corresponding raw sequencing data gave us the opportunity to investigate in detail the full complement of myticalin genes present in the genome of a single mussel specimen. Surprisingly, only four out of the 22 different precursor variants present in *M. galloprovincialis* transcriptomes (see Figure 1) were detected, indicating a lower than expected level of genomic complexity. While the absence of some myticalin genes could be potentially explained by the genome assembly fragmentation, these results were confirmed by multiple bioinformatics approaches, i.e., the independent reassembly of raw sequencing data using the CLC Genomics Workbench 10 (Qiagen, Hilden, Germany) and the mapping of the reads against full-length myticalin ORFs. The latter approach ruled out the possibility that missing myticalin genes were contained in unassembled genomic regions, as no read could be unambiguously mapped on such sequences, whereas the expected genome sequencing coverage was estimated to be ~30× [37].

Three of the genes identified pertained to group A (A3, A4 and A5) and a single gene pertained to group C (C10) (Table 1). As mentioned above, the genes A5 and C10 were complete, as they contained the three exons within the same contig, whereas the other myticalin genes were fragmented between two or three contigs. Neither the myticalin B1 gene nor any functional myticalin pertaining to group D could be found in the draft mussel genome. However, we could identify three myticalin pseudogenes with no evidence of expression. The first one showed 97% sequence identity to myticalin C3 and contained a nonsense mutation (confirmed by 100% mapped reads) within exon 2, expected to produce a truncated precursor. The second one showed 97% sequence identity to myticalin D7 and presented a large deletion within exon 2, expected to cause the loss of the signal peptide region. The correct assembly of the corresponding contig was confirmed by two assembly methods and by a consistent coverage of paired-end reads coverage. The third pseudogene, also pertaining to group D, did not share high similarity to any of the other myticalins and presented an ORF interrupted by two nonsense mutations within exon 2, fully confirmed by 100% mapped reads. The finding of multiple myticalin pseudogenes recalls a common feature of hypervariable gene families quickly evolving by gene duplication, such as those encoding toxins and defense peptides [34,38].

Overall, we report the presence of just four potentially functional myticalin genes pertaining to groups A and C in the draft mussel genome. Group D was represented by two pseudogenes and group B was not represented at all. These results suggest that considerable interindividual variability might occur in the inventory of myticalin genes across mussels, to the point that each individual could present a unique gene set, similarly to what has previously been reported for other AMPs in *Mytilus* spp. [5,39]. However, it remains to be established whether this variation is the result of extreme allelic variability linked to high heterozygosity [37], widespread genetic introgression within the *M. edulis* species complex [40] or whether it is linked to genome-wide phenomena of presence/absence variation, which have been previously described in oyster big defensins [41].

To test this hypothesis, we designed gene-specific primer pairs and preliminary tested the occurrence of different myticalins in 20 mussels, collected in the Gulf of Trieste (Italy) and belonging to the same natural population (Table S1). The successful amplification of myticalin C2, C5 and D1/D2 confirmed the presence of the corresponding genes in all the 20 tested genomes. On the other hand, in other cases we obtained an amplification product only in a few (C7) or in no mussel at all (C8), strongly hinting that these genes are not ubiquitously present. Further support of the presence/absence hypothesis was obtained with primers designed to co-amplify multiple myticalin genes, as a single or multiple amplification products (in particular for myticalin group A sequences), or no amplification at all (in some specimens, for myticalin D3/D4/D5) were evident.

Only the release of an improved assembly and annotation of the mussel genome and the resequencing of multiple individuals will provide definitive evidence for the possible involvement of presence/absence gene variability as a basis for the wide levels of interindividual variability of myticalins.

### 2.3. Myticalins Are Taxonomically Restricted to Mytiloida

Comparative genomics and transcriptomics analyses were employed to narrow down the distribution range of myticalins to a limited number of species pertaining to the order Mytiloida (Mollusca, Bivalvia). While no genome or transcriptome data is currently available for several minor lineages within the Bivalvia class, current data is certainly sufficient to exclude the presence of myticalins in non-mytiloid bivalves. Indeed, no myticalin-like sequence could be detected in the fully sequenced genomes of oysters and scallops [14,42,43], neither in the several dozen transcriptomes screened from Pteriomorphia, Heterodonta, Palaeoheterodonta, Archiheterodonta and Protobranchia (Table S2). Furthermore, neither BLAST nor HMM-based approaches led to the identification of significant similarities in non-bivalve mollusks and other metazoans.

**Figure 4:**
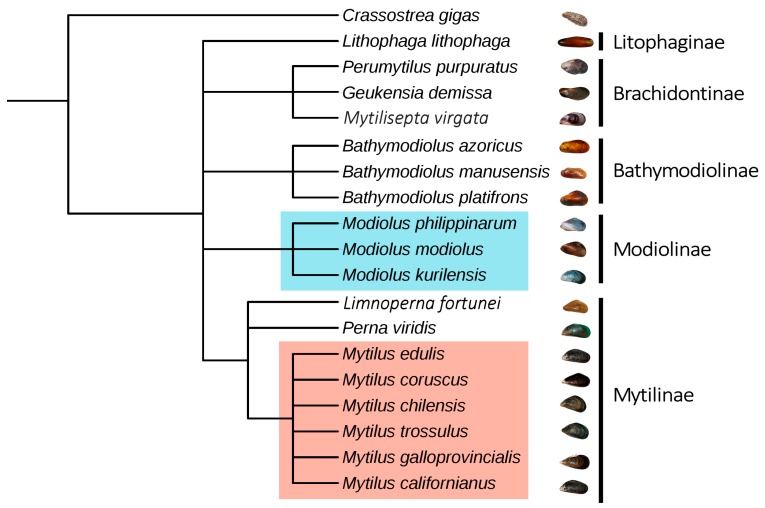
Taxonomic distribution of myticalins (light red) and modiocalins (light blue) within the order Mytiloida, as inferred by their presence/absence in the available transcriptomic and genomic sequence datasets. The Pacific oyster *C. gigas* was used as an outgroup to root the tree. The topology of the tree was retrieved from NCBI Taxonomy and the position of *M. virgata* was adjusted within Brachidontinae based on recent molecular data [44].

However, the analysis of available sequence data, indicated a patchy taxonomical distribution of myticalins even within the order Mytiloida, evidencing an apparent restriction to *Mytilus* and *Modiolus* spp. (Figure 4). Due to the marked divergence between *Mytilus* and *Modiolus* sequences, the latter were named modiocalins, as detailed in the next section. In detail, we detected myticalin sequences in all the interfertile species pertaining to the *Mytilus edulis* species complex (*M. galloprovincialis*, *M. edulis*, *M. trossolus* and *M. chilensis*) and, often, an identical sequence could be identified in multiple species, in agreement with the widespread genetic introgression already reported in this complex of species [40]. Myticalins of group A (A1, A2 and A6) or C (C4 and C7) were detected in *Mytilus californianus*, but no evidence of expression of myticalins B and D could be found in this species. Despite the relevant amount of RNA-seq data available for *Mytilus coruscus*, which include RNAs obtained from whole body, no full-length myticalin sequence was identified in the transcriptome of the Korean mussel.

The absence of myticalin-like genes in the genome of *Bathymodiolus platifrons* and in the transcriptomes of many other mussel species pertaining to Bathymodiolinae (*Bathymodiolus azoricus*, *Bathymodiolus manusensis*), Brachidontinae (*Mytilisepta virgata*, *Perumytilus purpuratus*, *Geukensia demissa*) and Litophaginae (*L. lithophaga*) strongly suggests that myticalin-related sequences are only present in some, but not all, mytiloids (Figure 4). The absence of myticalins in *Perna viridis* and *Limnoperna fortunei*, two members of the Mytilinae family together with *Mytilus* spp., further suggests that the evolutionary history of the myticalin gene family is complex and several independent gene loss events might explain such a taxonomic distribution. In any case, the lack of myticalin sequences within a transcriptome does not necessarily indicate, by itself, the absence of these genes, but rather just the lack of their expression. Only the future release of other mytiloid genomes and the analysis of novel sequence resources for other species will improve the preliminary overview depicted in Figure 4.

### 2.4. Modiocalins: Myticalin Homologs in Modiolus *spp.*

**Figure 5:**
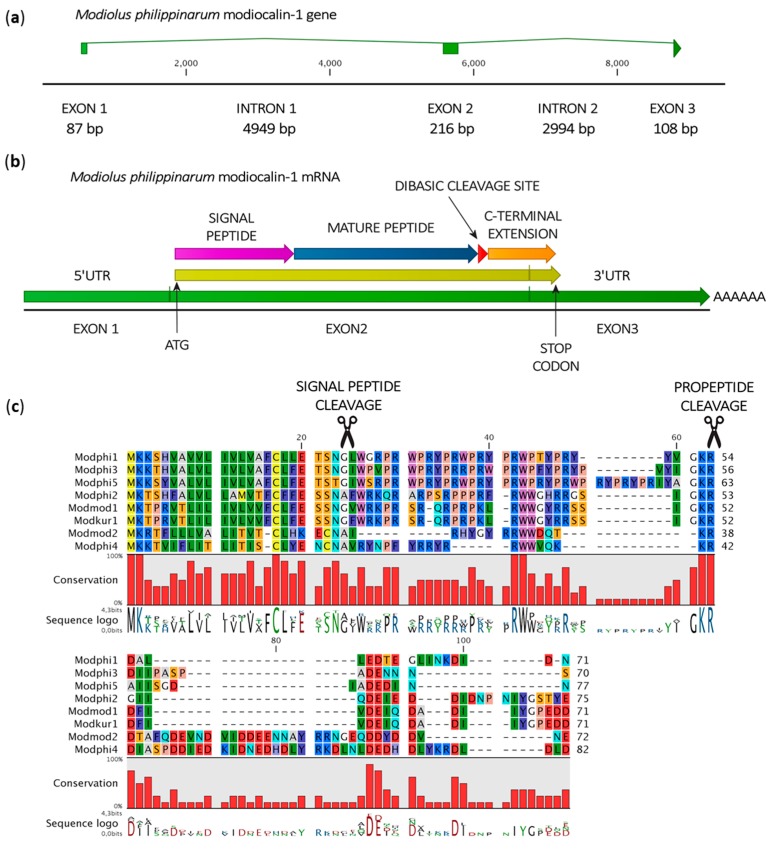
(**a**) Schematic organization of the *M. philippinarum* modiocalin-1 gene; (**b**) Organization of the *M. philippinarum* modiocalin-1 mRNA; (**c**) Multiple sequence alignment of *M. philippinarum* (Modphi), *M. modiolus* (Modmod) and *M. kurilensis* (Modkur) modiocalins (only full-length sequences with evidence of expression have been included).

The analysis of the recently released genome of *Modiolus philippinarum* [45] led to the identification of three potentially functionally complete myticalin-like genes (Mph_scaf_68178-0.0 and two unannotated genes located on scaffolds 10967 and 42638), a partial gene (unannotated, on scaffold 21923) and two pseudogenes containing in-frame STOP codons (both located on scaffold 33412). These sequences were named modiocalins (*Modiolus* cationic linear AMPs). Their evolutionary relationship with myticalins derive from several observations: (i) limited but still significant primary sequence similarity; (ii) identical gene architecture, including the presence of a phase 1 intron in the same position; (iii) identical protein precursor organization, containing a signal peptide, a central mature peptide region, a dibasic cleavage site and a C-terminal extension; (iv) high fraction of Pro and Arg residues in the mature peptide region; (v) prediction of high antimicrobial potential based on several in silico tools. In addition, modiocalins typically display a shorter and more variable C-terminal extension, which is nonetheless rich in anionic residues (Figure 5).

The sequence of the three full-length genes encoding modiocalin precursor proteins could be confirmed by the analysis of the *de novo* assembled transcriptome of *M. philippinarum*, which enabled their intron/exon boundaries to be defined in detail and the expression of the corresponding transcripts to be confirmed (tissue specificity could not be assessed since the RNA-seq data was obtained from a mix of tissues).

We detected modiocalins also in the gill transcriptome of the closely related species *Modiolus kurilensis:* a single functional sequence could be found in this case, while a second transcript contained an ORF interrupted by a premature STOP codon. Searches conducted in the *Modiolus modiolus* transcriptome (obtained from a mixture of gills, mantle and posterior adductor muscle) enabled the identification of a sequence identical at the amino acid level to that of *M. kurilensis* and a second one similar to *M. philippinarum* modiocalin-4 (Figure 5).

### 2.5. In Silico Evaluation of Antimicrobial Potential

Most of the 20 myticalins were identified as AMPs by a number of in silico predictor tools (Table S3). In detail, the antimicrobial character of myticalin mature peptides was predicted by CAMP3R [46] using three out of the four available prediction algorithms (80% positives with Discriminant Analysis, and Support Vector Machines, 75% positives with Random Forests). On the other hand, the CAMP3R prediction with Artificial Neural Network was negative in nearly 90% of cases.

With a single exception (myticalin C4), all mussel myticalins were identified as potential AMPS by iAMP-2L [47] and good results were also obtained with the Support Vector Machine approach of AntiBP2 [48] (75% positives) and with the sliding window approach of AMPA [49] (80% positives, not including myticalins of group D). The DBAASP predictor [50] was one of the few methods to only identify a very few myticalin sequences as potential AMPs. ClassAMP [51] marked all myticalins as antibacterial peptides with the Random Forests approach, whereas mixed results in terms of specificity (antibacterial, antiviral or antifungal) were obtained with the Support Vector Machine algorithm.

The majority of the applied prediction algorithms indicated most mussel myticalins as potentially bioactive peptides, with some differences among subfamilies. On average, group A myticalins were positively evaluated by 8 out of 10 methods, followed by groups C (7.5), B (6) and D (5.6) (Table S3). Myticalins A3 and C8 had the highest predicted antimicrobial potential (9 out of 10 methods).

Similarity searches with known AMPs listed in the Antimicrobial Peptides Database [52] revealed that the mature peptide region of myticalins did not share similarity higher than ~45% with any known AMP reported so far (Table S3). Class A myticalins displayed the highest homology to other AMPs (40–45%), in particular with arasin-2 expressed in the hemocytes of the small spider crab *Hyas araneus* [18], the apidaecin-based, proline-rich peptide A3-APO [53] and mammalian bactenecins (e.g., the cathelicidins PR-39 and OaBac5) [54,55]. However, these similarities are restricted to the mature peptide region, and the organization of the protein precursor of myticalins had no significant resemblance neither with cathelicidins nor with arasins.

We used a number of accessory algorithms to predict other aspects of the possible bioactive potential of myticalins. Following the conflicting indications obtained from the ClassAMP SVM algorithm, which indicated some myticalins as potential antiviral peptides, we tested this hypothesis with AVPpred [56], which only predicted a weak potential for this kind of activity. Furthermore, dPABBs predicted a negligible anti-biofilm potential for most myticalin peptides [57]. On the contrary, potential cell-penetrating activity upon interaction with cell membranes was assigned to all myticalins by CellPPD [58]. While the latter indication will require experimental validation, the in silico activity evaluation is confirmed as a convenient approach to preliminary define the possible mode of action of these mussel AMPs.

### 2.6. In Vitro Assessment of Antimicrobial Actvity

Seven peptides representative of the four main myticalin groups (Figure 2) were selected for solid phase synthesis and validation of the antimicrobial activity in terms of bacterial growth inhibition through minimum inhibiting concentration (MIC) assays. The selected peptides were tested against different Gram-positive and Gram-negative bacterial strains of environmental or medical relevance, not only to start exploring their spectrum of activity, but also to evaluate their potential as leading compounds for the development of new antibiotics (Table 2).

Myticalin D5, one of the peptides in silico predicted to have a low antimicrobial potential (Table S3), was the only one found to be completely inactive (MIC > 32 μM) toward all the tested bacterial strains. Two other peptides, myticalin B1 and C6, only displayed a moderate antimicrobial activity on a part of the tested strains. The other four myticalins had a remarkable antimicrobial activity, with significant MIC values (≤2 μM) against at least one of the tested bacterial strains, confirming the in silico prediction. Myticalins A5, A8, C9 and D2 displayed a broad spectrum of activity, being active against Gram-positive and -negative bacteria, although their potential activity in the natural marine environment remains to be ascertained. The spectrum of antibacterial activity of the Pro-rich myticalins A5, A8 and D2 interestingly matches that of well characterized linear Pro-rich peptides (PR-AMPs) previously identified in insects, crustacean and mammals [59], which are mainly active against Gram-negative species, especially *Escherichia coli*, *Acinetobacter baumannii* and, to a lesser extent, *Pseudomonas aeruginosa* [60,61], but also displayed a significant activity against the Gram-positive bacteria *Bacillus subtilis*. This selectivity derives from the fact that PR-AMPs act internally, and require a specific cytoplasmic membrane protein transport system to translocate to the cytoplasm, which is present in some Gram-negative bacterial species (e.g., *E. coli* and *A. baumannii*), but not in others (e.g., *P. aeruginosa*) or Gram-positive ones [60,61]. It is worth taking into account that a peculiar, lytic and not yet fully characterized mode of action has been suggested for PR-AMPs against *P. aeruginosa*, instead of the canonical non-lytic mechanism [62].

Myticalin C9 has a very high Arg content (32%) and is not rich in Pro residues. Nevertheless, it showed an antimicrobial potency to some extent comparable with Pro-rich group A and D myticalins, except from a significantly higher activity against *Staphylococcus aureus* (MIC = 2 μM).

None of the peptides inhibited the growth of *Vibrio anguillarum* ATCC 43305. The high NaCl content required in the medium for the growth of *V. anguillarum* is expected to reduce the binding of AMPs to bacteria and, therefore, their antimicrobial effect. On the other hand, it is also possible that *V. anguillarum*, similarly to other vibrios, has evolved intrinsic resistance mechanism towards the AMPs produced by their typical hosts [63].

### 2.7. Molecular Mode of Action of Myticalin A5

**Figure 6:**
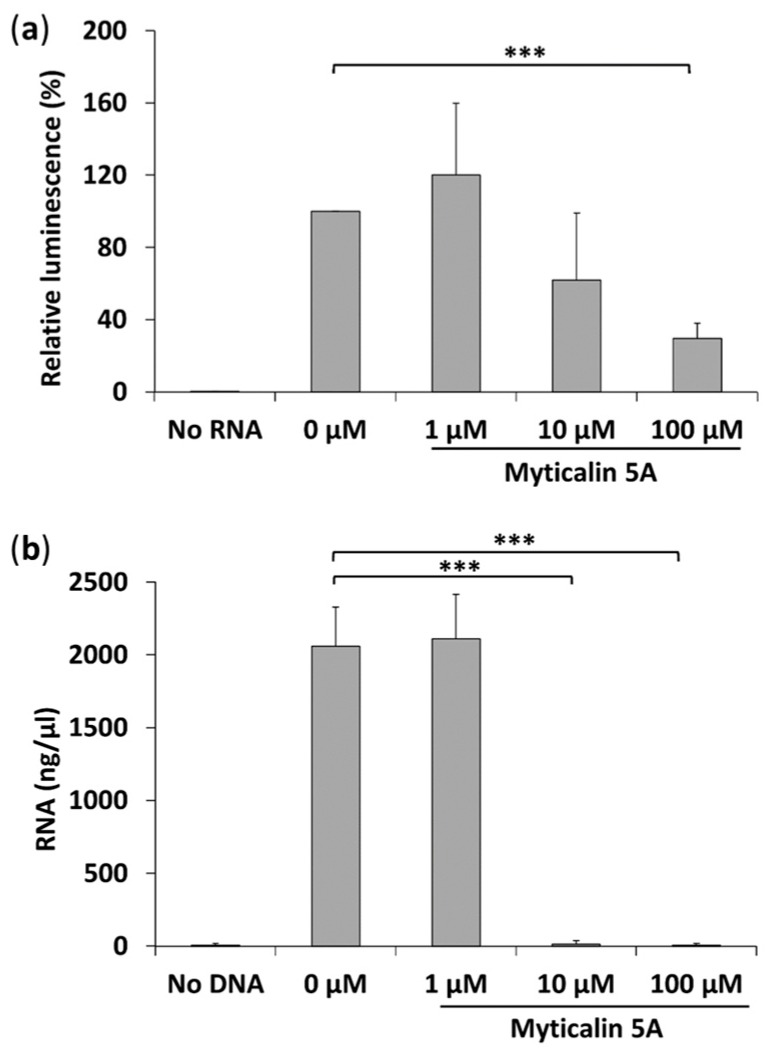
(**a**) In vitro translation and (**b**) in vitro transcription assay in the presence of myticalin A5. The positive controls received water instead of the peptide. The negative controls received water instead of the peptide and the DNA template encoding the luciferase. The results report the mean plus standard deviation of three independent experiments. *** indicate statistical significance *p*-value < 0.05, Student *t* test.

PR-AMPs can kill bacteria by inhibiting protein synthesis [64,65] by binding the ribosomal exit tunnel and impairing the elongation step of translation [66,67]. Based on these observations, we chose the mature peptide of myticalin A5, the richest one in terms of proline content (28%), to assess whether it could similarly inhibit protein synthesis in bacteria. As a first approach, a coupled in vitro transcription/translation reaction was performed in the presence of increasing peptide concentrations. The initial results indicated a moderate inhibition of the process (Figure S1). In subsequent assays aimed at dissecting the effects of the peptide on transcription and translation, the inhibiting effect exerted by the peptide on the translation reaction was surprisingly low (Figure 6a) and became significant only at 100 μM concentration. On the other hand, the peptide nearly completely inhibited the RNA synthesis, mediated by the T7 RNA polymerase used in the coupled transcription/translation reaction, at 10 μM concentration (Figure 6b).

Overall, it is reasonable to conclude that the moderate inhibition observed with the coupled in vitro transcription/translation assay was mainly due to an inhibition of the T7 RNA polymerase used in this kit to allow the high expression of the reporter gene. Therefore, myticalin A5 is unlikely to interact with the ribosomal tunnel, thereby blocking the protein synthesis process. The highly significant inhibiting activity on this peptide on the viral T7 RNA polymerase suggests that it might have an antiviral effect, despite the poor antiviral potential predicted by in silico methods (Table S3).

### 2.8. Tissue Specificity

The analysis of mussel transcriptome data suggested that, in contrast with most AMPs described so far in mussels [5,6,7], myticalins are preferentially expressed in the gills. Indeed, a number of myticalin transcripts could be *de novo* assembled to their full length using gill-derived RNA-seq. reads with relatively high sequencing coverage whereas they were only seldom detected in transcriptomes of other tissues.

**Figure 7:**
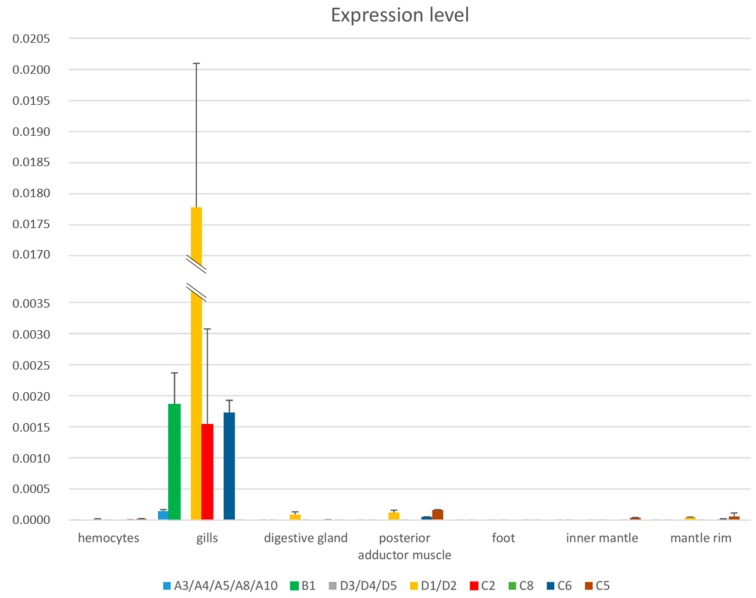
Tissue-specific expression of selected myticalins in *Mytilus galloprovincialis*, as detected by RT-PCR on samples pooled from 30 mussels. Bars represent the expression level normalized to the housekeeping gene elongation factor 1 alpha (*Y* axis). Error bars indicate the standard deviation of three technical replicates.

Following real time quantitative PCR (RT-PCR) assays, we fully confirmed this observation in adult unchallenged mussels: myticalins resulted to be mostly expressed in the gills and only barely detectable in hemocytes, digestive gland, adductor muscle and foot, inner and outer mantle (Figure 7). The expression level of myticalins A3/4/5/8/10, B1, C2, C6 and D1/D2 in gills was significantly higher than in the other tissues, whereas C5, C8 and D3/D4/D5 could only be detected at trace levels in various tissues. Altogether these results are consistent with the increasing recognition of the importance of mucosal immunity in marine bivalves [8,9]. Gills, in particular, represent the tissue with the broadest interface of interaction with potentially pathogenic microbes present in the water and conveyed into the intervalvar space by water filtration. The fibrillary structures of the gills are covered with a layer of mucus and they are often associated with complex bacterial communities which sometimes cover a function of the utmost importance in bivalve physiology [68,69].

It should be stressed out that myticalins maintained a limited expression level, as they remained in all cases less than 2% of the housekeeping gene EF1α (Figure 7). This observation is in stark contrast with the high constitutive expression of known AMPs in mussel hemocytes [70] and could be explained by different factors. First, the high interindividual sequence variability of myticalins and the possibility of genetic presence/absence variation, make it possible that primers designed on specific sequences may produce an amplification product only for a few of the mussels represented in a pooled sample. This would consequently lower the overall expression levels detectable for a specific AMP by RT-PCR in relatively large pools (*n* = 30). Second, we evaluated the myticalin expression levels in unchallenged animals, and we cannot rule out an upregulation in response to specific and still unknown pathogenic challenges. Considering these two factors, monitoring the expression of myticalins should ideally involve the sampling of gills before and after a pathogenic challenge in single individuals. Unfortunately, this approach is technically challenging, as it would undermine the health of animals, possibly resulting in their death.

## 3. Materials and Methods

### 3.1. Identification of Myticalin in the Mytilus Galloprovincialis Transcriptome

The previously described transcriptome of the Mediterranean mussel *M. galloprovincialis* [71] was subjected to an analysis with TransDecoder v.3.0.1 (http://transdecoder.github.io) to obtain virtual protein translations of any Open Reading Frame longer than 50 codons and shorter than 120 codons, a length interval which was selected based on the usual length of the precursor protein sequence of most AMP families found in mollusks and other invertebrates [72]. Only protein products potentially targeted to the secretory pathway were selected, based on the presence of a signal peptide region, identified with SignalP 4.1 [73]. Cationic AMP candidates were further selected based on their chemical-physical properties and amino acid composition, i.e., based on the presence of >30% arginine or proline residues over a sliding window of 15 amino acids, and/or isoelectric point >8 in a sliding window of the same length.

The sequences of the resulting candidate peptides were manually checked based on the assessment of a uniform read coverage by paired-end sequencing reads along the entire coding sequence, to exclude the possibility of misassembly. A target sequence, provisionally named myticalin (acronym for *Mytilus* cationic linear AMP), was selected due to the presence of an evident signal peptide, a central region satisfying the chemical-physical criteria mentioned above required for the detection of the mature peptide region, a Lys-Arg dibasic propeptide cleavage site clearly identifiable by ProP 1.0 [74] and a negatively charged C-terminal region, rich in aspartic and glutamic acid residues, a feature also shared by mussel defensins, mytilins and myticins [9].

### 3.2. Identification of Additional Members of the Myticalin Gene Family

Sequence similarity analyses by TBLASTN [75] revealed that myticalin belonged to a sequence family comprising many different members, which were detected based on an e-value threshold <1 × 10^−5^. Therefore, other publicly available transcriptomes from the same species (Table S2), were screened, with the aim of identifying all the existing sequence variants expressed in different tissues. The corresponding gene sequences, whenever available, were detected by TBLASTN against the mussel draft genome [37]. Exon and introns were detected by the alignment of assembled transcripts with genomic contigs and exon/intron junctions were refined with Genie [76].

Subsequently, the analysis was extended to other bivalve species, based on the dataset of transcriptomes described in [77] and the available genomes, i.e., *C. gigas* [42], *Pinctada fucata* [14], *Mizuhopecten yessoensis* [43], *Ruditapes philippinarum* [78], *L. fortunei* [79], *M. philippinarum* and *B. platifrons* [45]. A particular focus was pointed to the transcriptomes available for species pertaining to the order Mytiloida (Table S2), due to their close phylogenetic relationship with *Mytilus* spp. Another strategy also used as an alternative to TBLASTN, enabled to detect distantly related sequences. This was based on the search of an HMM profile, created staring from the MUSCLE multiple sequence alignment [80] of all *Mytilus* spp. myticalin sequences, performed with HMMER v.3.1b2 [81]. Due to the inherent limitation of current genome annotation pipelines for non-model metazoans [82], this approach was applied to the six-frame translations of all genomic scaffolds.

### 3.3. In Silico Prediction of the Antimicrobial Properties of Myticalins

The protein precursors resulting from the sequence search described above were analyzed with SignalP 4.1 [73] to identify signal peptide cleavage sites, and with ProP 1.0 [74] to identify dibasic proprotein convertase cleavage sites. We completed the prediction of mature peptide sequences by including the removal of C-terminal Arg or Lys residues by CPE and of C-terminal glycine residues by PAM, thereby resulting in C-terminally amidated peptides. Both molluscan enzymes are structurally and functionally similar to their vertebrate homologs and are involved in the post-translational modification of secreted peptides in gastropods [83,84].

The resulting predicted mature peptides were characterized by the calculation of isoelectric point with the ExPASy prediction servers (http://web.expasy.org/compute_pi/) and analyzed with a series of tools for the in silico prediction of antimicrobial activity. These included CAMPR3 (using the four available prediction algorithms, Random Forests, Discriminant Analysis, Support Vector Machines and Neural Artificial Network) [46], the predictor of the DBAASP database [50], AntiBP2 [48], AMPA [49], iAMP-2L [47] and ClassAMP (using both the Random Forests and Support Vector Machine algorithms) [51]. Furthermore, primary sequence homology with other known AMPs was investigated with APD3 [52]. The potential antibiofilm and antiviral activities of myticalins were predicted in silico with the dPABBs server (http://ab-openlab.csir.res.in/abp/antibiofilm/) [57] and AVPpred (http://crdd.osdd.net/servers/avppred/) [56], respectively. Finally, the cell-penetration activity of the mature peptides was predicted with CellPPD (http://crdd.osdd.net/raghava/cellppd/) [58]. The secondary structure was predicted with Porter [85].

### 3.4. Solid Phase Synthesis of Peptides

Seven myticalin peptides (A5, A8, B1, C6, C9, D2 and D5) were chosen on the basis of their distinctive sequences and mature peptide regions, predicted as described above. The first sequence was produced in the laboratories of the University of Trieste by solid phase synthesis using Fmoc chemistry on an Alstra automated microwave synthesizer (Biotage, Uppsala, Sweden). Upon positive preliminary functional tests, six additional peptide sequences were purchased from Synpeptide Co., Ltd. (Shangai, China). All peptides were purified by HPLC (Amersham Pharmacia Biotech, Milan, Italy) to a purity level >95%, and subsequently verified by MS analysis.

### 3.5. Bacterial Strains and Evaluation of the Antimicrobial Activity of Myticalins

The following strains have been used in this study: *Escherichia coli* ATCC 25922, *Staphylococcus aureus* ATCC 25932, *Vibrio anguillarum* ATCC 43305, *Pseudomonas aeruginosa* ATCC 27853, *Acinetobacter baumannii* ATCC 19606, *Bacillus subtilis* ATCC 6051. *Vibrio anguillarum* ATCC 43305 was cultivated in tryptic soy broth (TSB, Becton Dickinson, Milan, Italy) implemented with 2% NaCl, all the other strains were grown in Müller-Hinton broth (MH, Becton Dickinson, Milan, Italy).

The minimum inhibiting concentration (MIC) of each myticalin synthetic peptide was calculated using the broth microdilution method [86]. Briefly, twofold serial dilutions of each peptide were prepared in appropriate growth medium and aliquoted in the wells of a round-bottom microtiter plates (Sarstedt, Nümbrecht, Germany). An overnight culture of each bacterial strain was diluted 1:50 in fresh growth medium and grown to an OD_600nm_ ≈ 0.3. Each well was then inoculated with a standardized inoculum in order to achieve, in a final total volume of 100 μL, a final test concentration of about 5 × 10^5^ CFU/mL. The plate was then sealed to minimize evaporation and incubated at 37 °C for 24 h. The day after, the MIC was identified as the lowest concentration of the peptide that impaired any visible bacterial growth.

### 3.6. In Vitro RNA Synthesis, Purification and Quantification

To in vitro synthesize mRNA, in a final volume of 25 μL, 100 ng of a PCR template encoding firefly luciferase were mixed with 2.5 c of 10× T7 buffer, 100 mM NaCl, 80 mM MgCl_2_, 20 mM spermidine (Sigma Aldrich Chemie, Munich, Germany), 800 mM Tris-HCl (Sigma Aldrich Chemie, Munich, Germany), pH 8.0, DTT (Sigma Aldrich Chemie, Munich, Germany) to a final concentration of 5 mM, NTPs (ATP, GDP, UTP, CTP) (Sigma Aldrich Chemie, Munich, Germany) to a final concentration of 20 mM, 0.5 μL of RNAse inhibitor (RNAsin^®^, Promega, Madison, WI, USA) and 1 μL of recombinant custom-made T7 RNA polymerase (approx. 20 U). One μL of myticalin 5A solution at different concentrations was added to reach the concentrations of 1 μM, 10 μM and 100 μM. The positive control, received nuclease-free water instead of myticalin 5A. The negative controls, received nuclease-free water instead of myticalin 5A and DNA template. Samples were incubated for 2 h at 30 °C under agitation (300 rpm). Subsequently, all samples were treated with 0.5 U of TURBODNase (Thermo Fisher Scientific, Whaltam, MA, USA) to remove the DNA template, then centrifuged for 2 min, 12,000× *g* at 4 °C to remove the precipitated pyrophosphate. The cleared supernatants were mixed with LiCl (Sigma Aldrich Chemie, Munich, Germany) and EDTA (Sigma Aldrich Chemie, Munich, Germany) to the final concentration of 2.5 M and 17 mM, respectively, in a final volume of 92 μL. mRNA was precipitated by incubating samples for 1 h at −80 °C and by centrifugation for 30 min at 12,000× *g* and 4 °C. The RNA pellets were washed using 70% ethanol, samples were then centrifuged for 30 min, at 12,000× *g* and 4 °C, the ethanol was removed, the pellets were air dried for 5–10 min and resuspended in RNase-free water for RNA quantification using a NanoDrop 2000 (Thermo Fisher Scientific, Waltham, MA, USA) and for quality check by agarose-gel electrophoresis.

### 3.7. In Vitro Trasncription and Translation Assays

The in vitro translation was performed using the commercial kit RTS^TM^ 100 *E. coli* HY (Biotechrabbit, Hennigsdorf, Germany) following the instruction of the supplier. 0.1 μL of RNase Inhibitor (RNasin^®^, 20–40 U/μL, Promega, Madison, WI, USA) and 400 ng of synthetic mRNA coding for the firefly luciferase were added to the reaction mix in a final volume of 5 μL. The positive control received nuclease-free water instead of myticalin 5A. The negative controls received nuclease-free water instead of myticalin 5A and mRNA template. 1 μL of peptide solution was then added to the reactions get a final concentration of 1 μM, 10 μM or 100 μM. Samples were then incubated for 1 h at 30 °C with shaking (750 rpm). Subsequently 2 μL of each reaction were mixed with 8 μL kanamycin (50 mg/mL) to block any residual translation, and diluted with 40 μL of luciferase assays substrate (Promega, Madison, WI, USA) into a white flat bottom 96-well microtiter plate (Greiner Bio-One, Kremsmünster, Austria). The activity of the firefly luciferase was assessed and quantified using a Tecan Infinite M1000 plate reader. The reported relative luminescence data were calculated as a percentage of the positive control.

### 3.8. Evaluation of Gene Expression across Tissues

Primers were designed to assess the expression level of myticalins in the main tissues of adult unchallenged mussel specimens (hemolymph, digestive gland, posterior abductor muscle, inner mantle, mantle edge, gills and foot). Total RNA, extracted from 30 individuals of roughly the same size (5–7 cm) all sampled in the Gulf of Trieste (Italy), was quantified, pooled and reverse transcribed into cDNA like previously described [5].

Due to the relevant sequence similarity of many myticalin variants, some of the primer pairs designed (Table 3) allowed the simultaneous amplification of multiple highly similar targets. RT-PCR reaction were carried out on a C1000 thermal cycler (Bio-Rad, Hercules, CA, USA), using the following thermal profile: (i) initial denaturation at 95 °C for 3 min; (ii) 40 cycles at 95 °C for 5 s and 55 °C for 30 s. The lack of non-specific amplicons was assessed with a 65 °C/95 °C melting curve analysis. The PCR reaction, performed in a 15 μL volume, contained 7.5 μL SsoAdvanced™ SYBR^®^ Green Supermix (Bio-rad, Helcules, CA, USA), primers (0.3 μL each) and a 1:20 dilution of cDNA.

Gene expression levels, representing the mean plus standard deviation of three technical replicates, were calculated using the Δ*C*t method and normalized across tissues based on the housekeeping gene elongation factor 1 alpha, as described elsewhere [5,87].

### 3.9. Myticalin Gene Family Evolution

Due to the remarkable diversity of mature myticalin peptide sequences, only the coding part of the transcripts corresponding to the well-conserved signal peptide region and the C-terminal anionic extension (including the dibasic cleavage site and the two neighboring N-terminal residues) were considered for Bayesian phylogenetic inference. Briefly, nucleotide sequences were aligned with MUSCLE [80], maintaining codon triplets, and the resulting multiple sequence alignment was used to perform phylogenetic inference with MrBayes 3.2.6 [88], based on a codon w variation model. The analysis was run for one million generations, until all the estimated parameters of the model reached convergence. This was assessed with Tracer v.1.6 [89], making sure that all parameters reached an Effective Sample Size (ESS) value >200.

## 4. Conclusions

This study highlights the potential usefulness of in silico pipelines as complementary tools for AMP discovery in genomic and transcriptomic sequence data from non-model organisms. This strategy, with opportune modifications, appears to be particularly attractive not just for the discovery of AMPs, but also for the identification of other bioactive gene-encoded products in marine invertebrates, which can be considered as an invaluable source of natural compounds with potential biotechnological applications of great interest [90].

The indications obtained from the in silico prediction of antimicrobial potential of myticalins were confirmed by the positive outcome of microbiological assays, which revealed a potentially broad spectrum of activity against Gram-positive and Gram-negative bacteria of biomedical or environmental interest. However, these preliminary results need to be considered within the biological context where these AMPs are produced and are expected to exert their action. Taking into account the marked tissue specificity to the gills and the presence of a clear signal peptide for secretion, it seems reasonable that myticalins are released within the layer of mucus which covers the entire surface of this tissue, which represents the major interface of contact with the water column in filter-feeding bivalves and harbor of a complex microbiota [68,69]. Future studies could be focused on a better understanding of the biological targets of myticalins, to define whether they can be employed as routine microbiome-controlling agents or as specific killers of pathogenic bacteria.

Although the antibacterial activity of some myticalins could be attained at relatively low MIC concentrations (2–4 μM), the mode of action of these peptides remains to be elucidated. Like other PR-AMPs, myticalins were predicted to have relevant cell-penetrating properties, but preliminary tests have clearly shown that they are characterized by a divergent mode of action, as they are unlikely to inhibit protein synthesis by directly binding the bacterial ribosome. Only the detailed evaluation of the functional properties of myticalins will enable the development of biotechnological applications for these novel AMPs. At that stage, specific drug design strategies aimed at improving their stability and delivery in the target biological systems will need to be carefully taken into account [91,92].

## Figures and Tables

**Table 1:** Myticalin genes and pseudogenes detected in the *Mytilus galloprovincialis* draft genome.

Gene	Genomic Scaffold	Predicted Mature Peptide Sequence
Myticalin A3	8059-668075	YGWPRMPRIPRKPRYPRYPRYPRWPRHPTIYA-NH2
Myticalin A4	11814-355	YSWPRMPRIPRLPRYPRYPRYPRYPRWPRHPTIYA-NH2
Myticalin A5	371709-C71912861-576740	YSWPRMPRIPRLPRYPRYPRYPRWPRWPRQPTIYA-NH2
Myticalin C10	281970-C72301332	GRRRRYRYWRRGYRSWRRGVTIQERSKSSTLNTED
Myticalin C-PG	410031	RRRRRYRYWRRGLTI*GRSKSLPLNTGD ^1^
Myticalin D-PG1	8059	WGRRWRV*IPSPPRIRPWPP*TWPRPKWPRSATINID ^1^
Myticalin D-PG2	266177	WGRRLRIRIPSPPRPRPWPRPYPGPWPRSATINTDQ ^2^

PG: pseudogene. ^1^ virtual peptide, containing in frame STOP codons; ^2^ virtual peptide, with exon 2 partially deleted. * indicates nonsense mutations.

**Table 2:** Minimal inhibitory concentration (MIC) values for myticalins on selected bacterial strains.

Bacterial Species and Strain	Myticalin (μM)						
	A5	A8	B1	C6	C9	D2	D5
*Escherichia coli* ATCC 25922	2	1	8–16	4	4	4	>32
*Pseudomonas aeruginosa* ATCC 27853	4–8	8	>32	32	8	4	>32
*Acinetobcter baumannii* ATCC 19606	4	1	8	2	2	2	>32
*Vibrio anguillarum* ATCC 43305	>32	>32	>32	>32	>32	>32	>32
*Staphylococcus aureus* ATCC 25923	8–16	16	>32	8	2	16–32	>32
*Bacillus subtilis* ATCC 6051	4	4	32	32	4	2	>32

MIC values represent the mode of four independent experiments.

**Table 3:** Primers used for RT-PCR assays.

Sequence Name	Forward Primer (5′ → 3′)	Reverse Primer (5′ → 3′)
Myticalin A3/A4/A5/A8/A10	MGAATGCCACGGATACCAAG	TYATTCGTARMATCATCATTCA
Myticalin B1	GGAAGAAAACGAGCCACT	TCAATCCCGTCTCGCTCA
Myticalin D3/D4/D5	GGCRTTCTTTTGYTGTCAATG	GATCGTCGCCATGTTGGY
Myticalin D1/D2	TTTTYTTTGCWCTTTGTATGATGG	CGTCGTTCAAWATCGCTCG
Myticalin C2	GGCGACGAGGACTTACCATA	GCTCGTCGATATCGTCCATTA
Myticalin C8	AAGCCACAGATTGCCAAAAT	TCATCCATAACGTCCCCATT
Myticalin C6	GACGAAGGAGGCGAAGATTT	TCGTTCATCCKAAATGTCCKTC
Myticalin C5	TGAAAGGGTTTGTTTTGATGC	CAGTTCCTCTCTTTTGCGATG

## Supplementary Materials

The following are available online at www.mdpi.com/1660-3397/15/8/261/s1, Figure S1: in vitro coupled transcription/translation in presence of Myticalin A5; Table S1: Presence/absence patterns of myticalin genes in 20 mussel specimens collected in the gulf of Trieste, Italy; Table S2: list of transcriptomes screened for the presence of myticalins; Table S3: in silico prediction of myticalin biological activities.
