# Supplementary material for: Myticalins: A Novel Multigenic Family of Linear, Cationic Antimicrobial Peptides from Marine Mussels (Mytilus spp.)
Source: Mar Drugs. 2017 Aug 22;15(8):261. doi: 10.3390/md15080261 (PMC5577615; doi:10.3390/md15080261)
Supplement: Supplementary file 1 [file marinedrugs-15-00261-s001.pdf]

Supplementary Materials

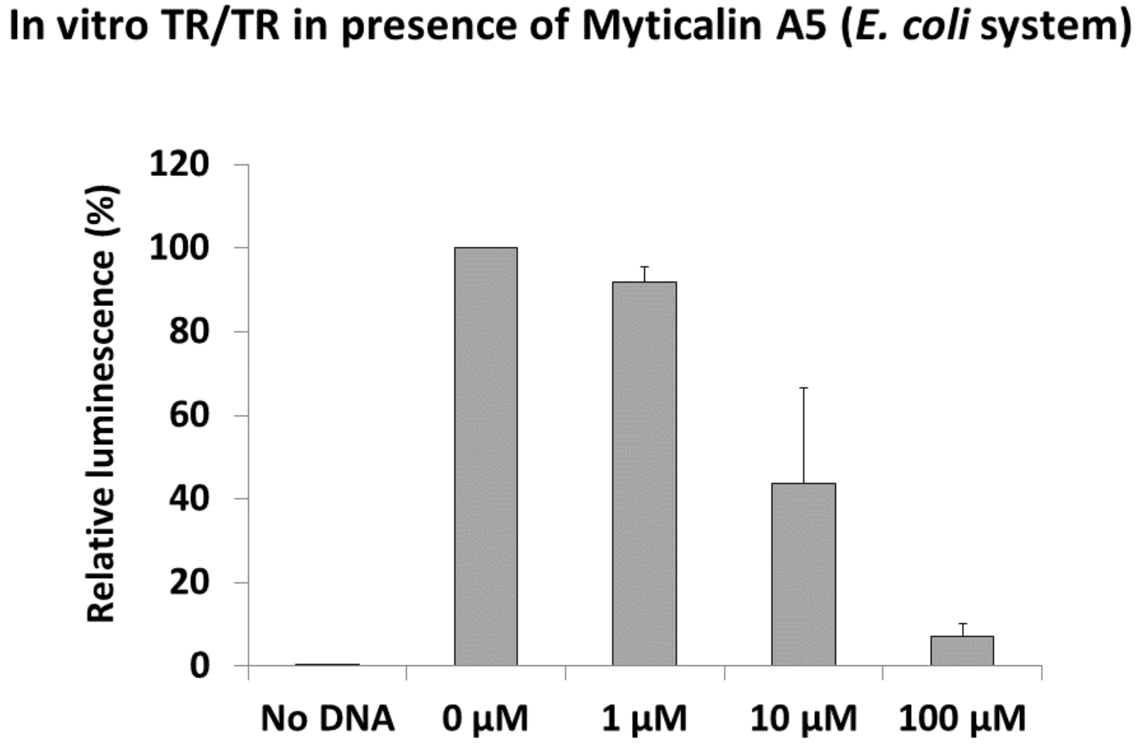

Figure S1. *in vitro* coupled transcription/translation in presence of Myticalin A5 in an *Escherichia coli* system. The positive control received water instead of the peptide. The negative control received water instead of the peptide and the DNA template encoding the luciferase. The results are the average of three independent experiments.

Table S1: Presence/absence patterns of myticalin genes in 20 mussel specimens collected in the Gulf of Trieste, Italy. ✓: present; ✕: absent; M: presence of multiple amplification bands.

	sample number																			
Myticalin	1	2	3	4	5	6	7	8	9	10	11	12	13	14	15	16	17	18	19	20
A3/A4/A5/A8/A10	M	M	✔	M	M	✔	✔	✔	M	M	M	✔	M	✔	✔	✔	M	M	M	✔
B1	✔	✔	✖	✖	✖	✔	✖	✖	✖	✖	✔	✖	✖	✔	✖	✖	✖	✖	✖	✖
C2	✔	✔	✔	✔	✔	✔	✔	✔	✔	✔	✔	✔	✔	✔	✔	✔	✔	✔	✔	✔
C5	✔	✔	✔	✔	✔	✔	✔	✔	✔	✔	✔	✔	✔	✔	✔	✔	✔	✔	✔	✔
C6	✖	✔	✔	✖	✖	✖	✖	✖	✖	✖	✖	✖	✖	✖	✖	✖	✖	✖	✔	✔
C8	✖	✖	✖	✖	✖	✖	✖	✖	✖	✖	✖	✖	✖	✖	✖	✖	✖	✖	✖	✖
D1/D2	✔	✔	✔	✔	✔	✔	✔	✔	✔	✔	✔	✔	✔	✔	✔	✔	✔	✔	✔	✔
D3/D4/D5	M	✔	✔	M	M	M	M	M	✖	✖	✖	✖	✔	✖	✖	M	M	M	✔	✔

Table S2. List of transcriptomes screened for the presence of myticalins.

***Mytilus* spp.**

Species	SRA datasets
Mytilus californianus	SRX565219-20
Mytilus chilensis	SRX1850488-9
Mytilus coruscus	SRR2895130-2, SRX792025, SRX791940
Mytilus edulis	SRX565221-4, ERR1414442-3, SRR1560431
Mytilus galloprovincialis	SRX126947-50; SRX565225-30; SRX1246876-7; SRX1240182; SRX389462-6; SRX389338; SRX386628
Mytilus trossulus	SRX565231-2

Other mytiloids

Species	SRA datasets
Bathymodiolus azoricus	DRR048281
Bathymodiolus platifrons	SRX1933530; SRX1933483; SRX610901-2; SRX436365
Bathymodiolus manusensis	SRX2481526-32
Geukensia demissa	kindly provided by prof. PA Fields
Lithophaga lithophaga	SRX1940727
Modiolus modiolus	SRX2357782
Modiolus kurilensis	SRX2481530-2
Modiolus philippinarum	SRX1934914-8
Mytilisepta virgata	SRX2497505; SRX2515148; SRX2515152; SRX2515284; SRX2515292
Perna viridis	SRX643367; SRX643380-5; SRX1873938-47
Perumytilus purpuratus	SRX2210805

Other bivalves

Species	SRA datasets
Anadara trapezia	SRX323049
Arctica islandica	SRX687761
Argopecten irradians	SRX470082
Astarte sulcata	SRX644667
Atrina rigida	SRX687763
Azumapecten farreri	SRX404364; SRX218546
Cardites antiquata	SRX687773
Cerastoderma edule	SRX687776
Corbicula fluminea	SRX209446
Crassostrea angulata	SRX481252-4
Crassostrea corteziensis	SRX641340; SRX471689
Crassostrea hongkongensis	SRX365659-60
Crassostrea virginica	SRX551498-516; SRX118365
Cycladicama cumingii	SRX687783
Cyrenoida floridana	SRX687777
Diplodonta sp. VG-2014	SRX701840

Donacilla cornea	SRX687785
Elliptio complanata	TSA: GAHW00000000.1
Ennucula tenuis	SRX091980
Eucrassatrella cumingii	SRX687772
Galeomma turtoni	SRX687768
Glossus humanus	SRX687780
Hiatella arctica	SRX687781
Lampsilis cardium	SRX687767
Lamychaena hians	SRX687779
Lasaea adansoni	SRX687778
Laternula elliptica	SRX1614611; SRX807593-8
Lyonsia floridana	SRX687774
Margaritifera margaritifera	SRX687769
Mercenaria campechiensis	SRX644681
Meretrix meretrix	SRX666741-4
Mizuhopecten yessoensis	SRX220583; SRX221165
Mya arenaria	SRX687786
Myochama anomioides	SRX644684
Neotrigonia margaritacea	SRX644685
Ostrea chilensis	SRX565248-9
Ostrea edulis	SRX565250-9
Ostrea lurida	SRX175407
Ostreola stentina	SRX565260-1
Pecten maximus	SRX497464
Phacoides pectinatus	SRX687782
Pinctada martensi	SRX268302
Placopecten magellanicus	SRX687766
Polymesoda caroliniana	SRX687784
Pyganodon grandis	SRX310669-84
Ruditapes decussatus	SRX348803-8
Ruditapes philippinarum	SRX076440-63
Saccostrea glomerata	SRX1884692-727; SRX1838009-12; SRX1797576-85; SRX964403-13; SRX878217; SRX845376
Solemya velum	SRX091478
Sphaerium nucleus	SRX687787
Uniomerus tetralasmus	SRX310669-97
Villosa lienosa	SRX101836-9
Yoldia limatula	SRX687760

Table S3. In silico prediction of myticalin biological activities.

	sequence of mature peptide	length	isoelectric point	CAMP SVM	CAMP Random Forest	CAMP Discriminant Analysis	CAMP Neural Artificial Network	DBA predictor	iAMP-2L	ANTIBP2 score	ANTIBP2 classification	AMPA	Class AMP - SVM	ClassAMP - SVM (classification)	ClassAMP-RF	ClassAMP - RF (classification)	ADP top hit	ADP details	AVP pred	dPABBs	cellPPD	Pro%	Arg%	Trp%	Tyr%	Thr%
A1	IGWPRFPRLPRTPRYPRYPRYPTWPTYPRYPSWPRYA-NH2	37	11.29	0.99	0.705	0.849	NO	NO	YES - antibacterial	0.291	Insect - lebocin	83%	94%	antiviral	85%	antibacterial	42.85%	cathelicidin PR39	1	NO	CPP	29%	21%	8%	16%	8%
A2	IGWPRFPRLPRTPRYPRYPRYPRWPTYPRYPSWPRYA-NH2	37	11.51	0.997	0.719	0.903	NO	NO	YES - antibacterial	0.304	Insect - lebocin	89%	94%	antiviral	87%	antibacterial	45.23%	cathelicidin PR39	1	NO	CPP	29%	24%	8%	16%	5%
A3	YGWPRMPRIPRKPRYPRYPRYPRWPRHPTIYA-NH2	32	11.57	0.975	0.512	0.878	NO	YES	YES - antibacterial	0.413	Insect - lebocin	95%	94%	antiviral	94%	antibacterial	42.42%	A3-APO	1	NO	CPP	28%	25%	6%	15%	3%
A4	YSWPRMPRIPRLPRYPRYPRYPRYPRWPRHPTIYA-NH2	35	11.51	0.992	0.558	0.697	NO	NO	YES - antibacterial	0.305	Insect - lebocin	94%	93%	antiviral	93%	antibacterial	42.22%	Arasin-2	1	NO	CPP	28%	25%	5%	17%	2%
A5	YSWPRMPRIPRLPRYPRYPRYPRWPRWPRQPTIYA-NH2	35	11.72	0.982	0.541	0.424	NO	NO	YES - antibacterial	0.399	Insect - lebocin	94%	99%	antibacterial	95%	antibacterial	43.47%	Arasin-2	1	NO	CPP	28%	25%	8%	14%	2%
A6	YSWPRLPRIPRLPRYPRYPRWPRHPTIYA-NH2	29	11.63	0.977	0.694	0.732	NO	NO	YES - antibacterial	0.428	Insect - lebocin	89%	95%	antifungal	96%	antibacterial	44.82%	A3-APO	1	NO	CPP	27%	24%	6%	17%	3%
A7	YSWPRLPRIPRLPRYPRYPRYPRWPRHPTIYA-NH2	32	11.56	0.993	0.702	0.742	NO	NO	YES - antibacterial	0.357	Insect - lebocin	92%	94%	antifungal	95%	antibacterial	42.42%	A3-APO	1	NO	CPP	28%	25%	6%	15%	3%
A8	YSWPRMPRIPRLPRYPRYPRYPRWPRHPTIYA-NH2	32	11.56	0.973	0.515	0.69	NO	NO	YES - antibacterial	0.391	Insect - lebocin	92%	95%	antibacterial	95%	antibacterial	42.42%	A3-APO	1	NO	CPP	28%	25%	6%	15%	3%
A9	YSWPRIPRIPRLPRYPRYPRYPRYPRWPRHPTIYA-NH2	35	11.51	0.997	0.705	0.837	NO	NO	YES - antibacterial	0.309	Insect - apidaecin	93%	94%	antiviral	93%	antibacterial	43.18%	cathelicidin OaBac5	1	NO	CPP	28%	25%	5%	17%	2%
B1	LRWTPTPSYPRYPTRSRGSRWSR-NH2	23	12.01	0.201	0.621	0.977	NO	NO	YES - antibacterial, antifungal	-0.445	/	100%	95%	antiviral	68%	antibacterial	40%	A3-APO	0	NO	CPP	17%	26%	8%	8%	13%
C1	GRRRKYRYWRRGDRYWRRGVTIQERSKSSTLNTED	35	11.44	0.697	0.466	0.962	NO	NO	YES - antibacterial	0.189	Mammals - cathelicidin	100%	89%	antifungal	68%	antibacterial	35%	rhesus macaque myeloid alpha-defensin-6	1	YES	CPP	0%	28%	5%	8%	8%
C2	GRRRRRYRYWRRGLTIQGRSSTTITGD	27	12.1	0.619	0.508	0.978	NO	NO	YES - antibacterial, antifungal	-0.119	/	100%	92%	antiviral	71%	antibacterial	37.50%	chicken cathelicidin 2	1	NO	CPP	0%	33%	3%	7%	14%
C3	RRRRRYRYWRRGLTIQGRSKSLPLNTGD	28	12.1	0.65	0.467	0.91	NO	NO	YES - antibacterial, antifungal	0.168	Mammals - cathelicidin	100%	93%	antiviral	79%	antibacterial	37.83%	rhesus macaque myeloid alpha-defensin-6	1	NO	CPP	3%	32%	3%	7%	7%
C4	RRRRRYRYWRRGVTIQGRSKYSTLNTGE	28	11.91	0.711	0.458	0.878	NO	NO	NO	0.091	Mammals - cathelicidin	100%	93%	antiviral	65%	antibacterial	36.83%	rhesus macaque myeloid alpha-defensin-6	1	YES	CPP	0%	32%	3%	10%	10%
C5	RRRRWPRRVTRRIRIPRYLTLNTH	24	12.6	0.997	0.67	0.996	YES	NO	YES - antibacterial, antifungal	-0.138	/	100%	98%	antiviral	97%	antibacterial	39.28%	WLBU2	1	YES	CPP	8%	41%	4%	4%	12%
C6	RRRRRFRRVIRRIRLPKYLTINTE	24	12.37	0.997	0.637	0.993	YES	NO	YES - antibacterial, antifungal	-0.043	/	100%	93%	antiviral	82%	antibacterial	41.37%	chicken cathelicidin 2	1	YES	CPP	4%	41%	0%	4%	8%
C7	RRRRWRRIRRGISIRLPKFATLNTE	25	12.54	0.981	0.719	0.984	YES	NO	YES - antibacterial, antifungal	-0.007	/	100%	97%	antiviral	89%	antibacterial	39.28%	NRC-3	1	YES	CPP	4%	36%	4%	0%	8%
C8	RRRRWRRIRRGISHRLPKFATLNSD	25	12.54	0.935	0.593	0.983	YES	YES	YES - antibacterial, antifungal	-0.089	/	100%	95%	antiviral	90%	antibacterial	35.71%	NRC-3	1	YES	CPP	4%	36%	4%	0%	4%
C9	RRRRRYRYWRRGLTIQGRPKSLPLNTGD	28	12.1	0.722	0.495	0.903	NO	NO	YES - antibacterial, antifungal	0.015	Mammals - cathelicidin	100%	94%	antiviral	81%	antibacterial	36.84%	BG-CATH37	2	YES	CPP	7%	32%	3%	7%	7%
C10	GRRRRYRYWRRGYRSWRRGVTIQERSKSSTLNTED	35	11.77	0.602	0.501	0.984	NO	NO	YES - antibacterial	0.039	Mammals - cathelicidin	100%	89%	antifungal	74%	antibacterial	36.58%	rhesus macaque myeloid alpha-defensin-6	1	NO	CPP	0%	31%	5%	8%	8%
D1	WGRRWRIRIPSPPRLRPWPRPYPRPWPRSATINTDQ	36	12.3	0.509	0.681	0.455	NO	NO	YES - antibacterial	0.346	Frog - other	0%	99%	antifungal	94%	antibacterial	41.60%	cathelicidin OaBac5	1	NO	CPP	25%	25%	11%	2%	5%
D2	WGRRWRIRIPRLPRPWPPRPKWPRSATINTDQ	32	12.48	0.738	0.699	0.726	NO	NO	YES - antibacterial	0.146	Frog - other	99%	99%	antifungal	96%	antibacterial	39.02%	CRS4C-2	1	NO	CPP	21%	25%	12%	0%	6%
D3	WPRFPKPRKPTYSGPTYPGPTYPRPTLPRPTWRRSATIGTDH	42	11.4	0.321	0.405	0.013	NO	NO	YES - antibacterial	0.007	Frog - other	0%	99%	antifungal	81%	antibacterial	38.29%	Gm pro-rich pept 2	1	NO	CPP	26%	14%	4%	7%	16%
D4	WPRFPKPRKPTYPGPTYPGPTWPRPTWPRPTWRRSATIDTEH	42	11.38	0.345	0.419	0.008	NO	NO	YES - antibacterial	0.117	Frog - other	0%	99%	antifungal	74%	antibacterial	37.25%	cathelicidin OaBac5	1	NO	CPP	28%	14%	9%	4%	16%

D5	WPRFPKPRKPTYPGPTYPGP TWPRPTWRRSATIDTEH	37	10.95	0.235	0.371	0.015	NO	NO	YES - antibacterial	0.106	Frog - other	0%	99%	antibacterial	73%	antibacterial	37.20%	arasin-2	1	NO	CPP	27%	13%	8%	5%	16%
D6	WRRRWRVRIPWPPRPRWP RSATINTDQ	27	12.48	0.495	0.631	0.841	NO	NO	YES - antibacterial	-0.018	/	99%	97%	antiviral	90%	antibacterial	37.03%	apidaecin	1	YES	CPP	18%	29%	14%	0%	7%
D7	WGRRWRIRIPSPPRPRPWP RPYPRPWPRSATINTDQ	36	12.3	0.52	0.679	0.63	NO	NO	YES - antibacterial	0.529	Frog - other	0%	98%	antifungal	94%	antibacterial	43.75%	cathelicidin OaBac5	1	NO	CPP	27%	25%	11%	2%	5%
Mp1	LWGRPRWPRYPRWPRYPR WPTYPRYYI-NH2	27	11.33	0.985	0.706	0.917	NO	NO	YES - antibacterial	0.018	Frog - other	0%	91%	antiviral	88%	antibacterial	44.44%	A3-APO	1	NO	CPP	25%	25%	14%	18%	3%
Mp2	FWRKQRARPSRQPPRFRW WGNRRGSI-NH2	26	12.85	0.923	0.704	0.992	YES	NO	YES - antibacterial	-0.142	/	100%	97%	antiviral	86%	antibacterial	39.39%	Melimine	0	YES	CPP	11%	30%	11%	0%	0%
Mp3	IWPVPRWPRYPRRPRWPR WPFYPRYPVYI-NH2	29	11.63	0.951	0.72	0.758	NO	NO	YES - antibacterial	0.292	Frog - other	93%	97%	antifungal	89%	antibacterial	44.66%	A3-APO	1	NO	non-CPP	31%	24%	13%	13%	0%
Mp4	VRYNPFYRRYRRWWVQ	16	11.45	0.294	0.531	0.856	NO	YES	YES - antibacterial	0.002	Frog - other	0%	88%	antibacterial	81%	antibacterial	36.84%	Astacidin 2	1	YES	non-CPP	6%	31%	12%	18%	5%
Mp5	FWVRPRWPRYPRWPRYPR WPRYPRWPRYPRYPRIYA-NH2	36	11.77	1	0.72	0.973	NO	NO	YES - antibacterial	0.037	Frog - other	0%	89%	antiviral	70%	antibacterial	44.18%	PR-39	1	NO	CPP	27%	30%	13%	16%	0%
Mk1/Mm1	FWRKPRSRQRPRPKLRWW GYRRSSI-NH2	25	12.48	0.509	0.673	0.993	YES	NO	YES - antibacterial, antifungal	-0.104	/	0%	97%	antifungal	90%	antibacterial	36.00%	Astacidin 2	1	YES	CPP	12%	32%	12%	4%	0%
Mm2	IRHYGYRRWWDQT	13	9.98	0.071	0.439	0.094	NO	NO	NO	ND	/	93%	78%	antiviral	71%	antibacterial	40.00%	Odorranain-T1	0	YES	non-CPP	0%	23%	15%	15%	7%

Mp *Modiolus philippinarum*
Mm *Modiolus modiolus*
Mk *Modiolus kurilensis*
